# Dimethyl Sulfide (DMS) in Amarone Wines: Influence of Aging, Withering, Grape Variety, and Geographical Origin

**DOI:** 10.1021/acs.jafc.3c00728

**Published:** 2023-04-21

**Authors:** Jessica A. Samaniego Solis, Giovanni Luzzini, Davide Slaghenaufi, Maurizio Ugliano

**Affiliations:** Department of Biotechnology, University of Verona, Villa Lebrecht, via della Pieve 70, 37029 San Pietro in Cariano, Italy

**Keywords:** dimethyl sulfide (DMS), wine aging, Amarone wine, withering, terroir

## Abstract

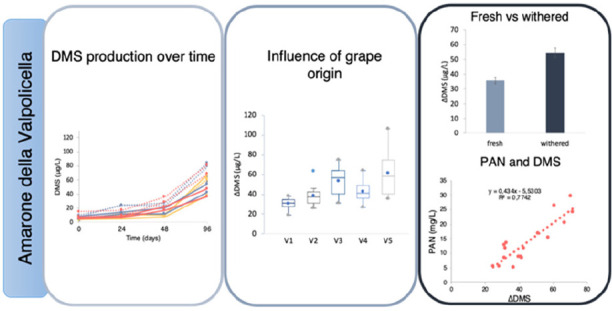

Occurrence of dimethyl sulfide (DMS), a potent aroma compound accumulating during aging, was investigated in commercial and experimental Amarone wines. In commercial Amarone, DMS was observed in concentrations ranging from 2.9 to 64.3 μg/L. Model aging studies on experimental wines indicated that DMS in Amarone is strongly associated with aging and that wines from different vineyards can vary significantly in their ability to accumulate DMS during aging. The capacity of certain vineyards to give wines with higher DMS-forming potential was consistent across three consecutive vintages, representing a true terroir factor to be expressed with aging. Wine content of primary amino acids (PAN), a commonly analyzed enological parameter of grape must, was shown to be positively correlated with DMS accumulation during aging. Grape withering also increased DMS-forming potential mostly due to increased PAN resulting from concentration due to water loss. Increased pH due to withering also contributed to a higher DMS content of withered wines, but to a lower extent. In certain vineyard sites, an influence of vintage conditions on DMS-forming potential was also observed.

## Introduction

Dimethyl sulfide (DMS) is a low molecular weight sulfur-containing compound found in wine, which has attracted noticeable interest due to its complex chemistry and biochemistry as well as for its potential sensory contribution, in particular in aged red wines.^1−4^

The presence of DMS in wine is often associated with odor notes of asparagus, cauliflower, canned corn, black olives, and truffle.^2,5,6^ The reported threshold of DMS is 10 μg/L in 10% (v/v) ethanol solution^7^ and 25–60 μg/L in red wine,^8−10^ and the type of sensory contribution can vary significantly depending on concentration and wine matrix composition. Relatively low concentrations of DMS (10 μg/L) can support the expression of sweet tobacco, fruity, and strawberry attributes in combination with esters and norisoprenoids,^3^ while higher concentrations (50–100 μg/L) are associated with black olive and truffle odors.^1,3,11^ In another study, DMS was found to contribute positively to wine aroma, enhancing the expression of black fruit and in particular blackcurrant aromas when present in combination with other fruity compounds such as esters and β-damascenone.^1^

Unlike other wine sulfur compounds, which are mostly associated with fermentation and/or attain maximum concentration during or at the end of fermentation,^12^ DMS content of young wines is typically low and in most cases of negligible sensory relevance. Conversely, aged wines are characterized by much higher levels of DMS, resulting from hydrolysis of precursors present in the wine.^13^*S*-Methylmethionine (SMM), a derivative of methionine, has been reported as one of the main precursors of DMS.^2,14,15^ Many factors modulate the occurrence of DMS in wine and of its precursors in grape and wine, including grape cultivar, viticultural and winemaking practices, vineyard nitrogen status, wine pH, and storage temperature conditions.^16−18^ Because the occurrence of sensorially relevant DMS levels in wine is essentially related to aging,^1^ understanding the relationship between DMS in aged wine and precursor content of both grapes and young wine can improve the capacity to tailor wine aging aroma potential. To this point, analytical approaches based on the quantification of potential DMS by means of harsh alkaline treatment of grape extracts or wine have been used to investigate the relation with potential DMS and grape variety,^11^ vineyard water status,^17^ and YAN levels.^18^ Conversely, studies addressing the evolution of free DMS during actual or accelerated wine aging have been less frequent, although it has been shown that this compound can form under conditions of mild accelerated aging at natural wine pH.^19^

Most studies concerning DMS in aged wines have concentrated on red wines from French native grape varieties such as Grenache, Syrah, and blends of Cabernet-Sauvignon with Merlot,^11,12,18^ as well as on sparkling white wines.^20^ Amarone della Valpolicella is an Italian red wine produced in the Valpolicella area, in northeastern Italy, that has gained considerable attention in the last two decades. The peculiarity of Amarone is that it is one of the very few dry red wines obtained from partially withered grapes, primarily *Vitis vinifera* L. *cv*. Corvina and *cv*. Corvinone. The central role of withering in determining unique metabolic profile of withered grapes and wines has been shown for terpenes, norisoprenoids, and phenolic compounds,^21,22^ while other studies have investigated the influence of variety, grape geographical origin, and fermentation conditions in Valpolicella wines volatile composition.^23−26^ Fedrizzi et al.^27^ reported significant increases in DMS content of Amarone during accelerated aging experiments, confirming the presence of DMS precursors. The appellation regulation concerning Amarone production indicates a minimum aging period of two years before market release,^28−30^ although most producers opt for 4–5 years of cellar aging before releasing the wines. Accordingly, chemical reactions that are relevant to aroma evolution during aging are of interest for Amarone aroma composition at the time of its release. Anecdotal evidence also refers often to berry and black currant aromas as one of the primary Amarone aroma attributes.

The main aim of the present work was to investigate the occurrence of DMS in commercial Amarone wines and elucidate its association with some of the major factors associated with Amarone production, namely, aging, withering, variety, and geographical origin of the grapes. Throughout the work, an accelerated aging protocol was used, where the relationship between wine compositional and production variables and evolution of wine actual DMS content will be explored.

## Materials and Methods

### Amarone Commercial Wines

Thirty-two Amarone commercial wines were used for the present study: 17 wines from vintage 2015 and 15 from vintage 2016. Wines were obtained from different wineries in the Valpolicella area and were analyzed in the spring of 2020 and 2021 for the 2015 and 2016 vintages, respectively. For each wine, two bottles of the same production lot were obtained and were pooled prior to analysis. The following pairs were wines of the same winery from the two different vineyards: AM01–AM23, AM15–AM28, AM02– AM19, AM16–AM29, AM03–AM20, AM17–AM18, AM05–AM22, AM07–AM32, AM09–AM31, AM12–AM21, and AM14–AM30. Wine enological parameters for these wines are given in Supporting Information S1.

### Influence of Vineyard of Origin, Withering, Grape Variety, and Vintage on DMS Formation during Aging

Sixty experimental wines produced with Corvina (*Vitis vinifera* L. cv. Corvina) and Corvinone (*Vitis vinifera* L. *cv*. Corvinone) grapes were used for this study. Grapes were harvested during three vintages (2017, 2018, and 2019) from five different vineyards belonging to the same winery (Supporting Information S2). The location of the vineyards corresponded to two subregions within Valpolicella. Vineyards 1–3 were located between the municipalities of Tregnago and Mezzane di Sotto (45°30′36.7″N 11°08′02.7″E, reference weather station Illasi,; while vineyard 4 was located in Pedemonte (45°30′40.5″N 10°54′58.6″E, reference weather station San Pietro in Cariano) and vineyard 5 in San Giorgio in Valpolicella (45°32′16.6″N 10°51′19.4″E, reference weather station Marano in Valpolicella). Temperature and rainfall conditions of the three vintages are given in Supporting Information S3. Harvest dates were as follows: vintage 2017, 13th to 24th September; vintage 2018, 17th September to first October; and vintage 2019, 25th September to 14th October. Information related to the technological maturity of the grapes can be found in Supporting Information S4. Vinification of fresh grapes was carried out at harvest, whereas, for withering, a portion of the fresh grapes was placed in a warehouse for 11–12 weeks, until a 30% of weight loss was reached. The warehouse conditions, where the withering was carried out, showed a gradual temperature decrease from 16 to 7 °C and a progressive increase in relative humidity from 55% to 80%. All vinifications were carried out in triplicate, as previously described.^31^

The model aging protocol proposed by Luzzini et al.^31^ was followed with some modifications. Wines bottles were opened and under a gentle N_2_ stream poured into glass vials with aluminum crimp closure to a final volume of 60 mL without headspace and then sealed off with epoxy resin. Aliquots of the different wines were placed at 45 °C for 24, 48, and 96 days. Simultaneously, control samples were stored at 4 °C. All samples were prepared in triplicate and analyzed by headspace solid-phase microextraction (HS-SPME) coupled to gas chromatography–mass spectrometry (GC–MS) following the method of Slaghenaufi et al.^32^

### Influence of pH on DMS Formation during Aging

An Amarone wine from vintage 2015 (initial concentration of DMS: 10.3 μg/L and pH 3.45) was spiked with *S*-methylmethionine (SMM) in the form of dl-methionine methylsulfonium chloride (Merck, Darmstadt, Germany) at 6.4 mg/L, in order to have a final concentration of 2 mg/L of DMS equivalents.^2^ Aliquots of the spiked wine were then prepared as follows: (i) samples of wine nonspiked at pH 3, (ii) samples of wine nonspiked at pH 4, (iii) samples spiked at pH 3, and (iv) samples spiked at pH 4. The pH adjustments were carried out by adding a sodium hydroxide solution 1 M (Honeywell, Seelze, Germany). Samples were poured under a N_2_ stream into glass vials with aluminum crimp closure and sealed off with epoxy resin. Samples were prepared in triplicate and put at 45 °C for 1 week and one month. All experiments were performed in triplicate.

### DMS Analysis by HS-SPME–GC–MS

DMS in both commercial Amarone wines and wines submitted to model aging were analyzed using HS-SPME–GC–MS following the method of Slaghenaufi et al.^32^ Ten milliliters of wine was transferred into 20 mL vials containing 3 g of NaCl and spiked with 100 μL of internal standard DMS-*d*_6_ (2 mg/L in ethanol). Samples were equilibrated at 35 °C for 5 min; then, a PDMS-DVB SPME fiber was exposed into the headspace of the sample for 30 min. Desorption in the injector was performed at 270 °C for 7 min. Gas chromatography analysis was performed using a HP 7890A (Agilent Technologies, Santa Clara, CA) coupled to a 5977B mass spectrometer equipped with an auto sampler (Gerstel MPS3, Mülheim/Ruhr, Germany). Injection was performed in splitless mode. Separation was done using a DB-WAX UI capillary column (30 m × 0.25 mm i.d., 0.25 μm film thickness, Agilent Technologies) with helium (6.0 grade) used as a carrier gas at 1.0 mL/min constant flow rate. The oven temperature program was set with the following conditions, starting at 35 °C for 5 min, raised to 90 °C at 5 °C/min, and then raised again to 240 °C at 10 °C/min and kept for 5 min. A mass spectrometer was operated in electron ionization (EI) at 70 eV with the ion source temperature set at 250 °C and quadrupole temperature at 150 °C. SIM mode was used for mass spectra acquisition. Calibration curves were obtained using Chemstation software (Agilent Technologies, Inc.) by linear regression plotting response ratio (analyte peak area divided by peak area of internal standard) against concentration ratio (added analyte concentration divided by internal standard concentration). The limit of detection and limit of quantification of the method were 0.02 and 0.06 μg/L respectively, while the linearity range was 0.06–200 μg/L.

### Grape and Wine Enological Analyses

Primary amino nitrogen (PAN) content of grapes and wines was obtained using a Biosystems Y15 multiparametric analyzer (Sinatech, Fermo, Italy). The method is based on amino acid derivatization with *o*-phthaldialdehyde (OPA). The pH of the wines was acquired with a Crison Basic 20+ pH meter (Crison, Barcelona, Spain).

### Statistical Analyses

Kruskal–Wallis test (α = 0.05) and Spearman correlation test (α = 0.05) were performed using XLSTAT 2017 (Addinsoft SARL, Paris, France).

## Results

### DMS Occurrence in Amarone Commercial Wines

**Figure 1:**
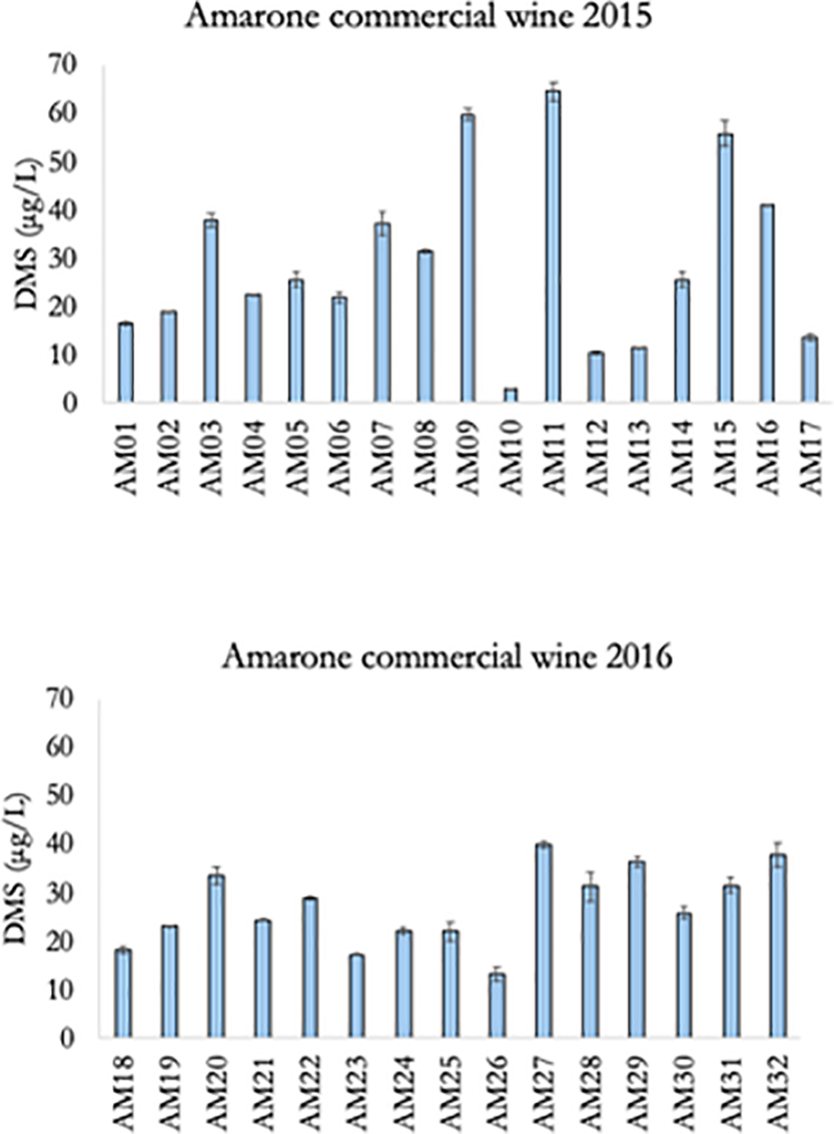
DMS concentrations (μg/L) in Amarone commercial wines from vintages 2015 and 2016.

The data concerning the DMS content in Amarone wines is shown in Figure 1. Across the two vintages, an average concentration of DMS of 27.9 μg/L was found. Samples from 2015 showed DMS content up to 64.3 μg/L, although samples with values lower than 5 μg/L were also observed. Wines of the 2016 vintage showed less variability, with maximum concentrations being lower than those in 2015 (highest value 39.8 μg/L), although no sample with a DMS content lower than 13.3 μg/L was observed. Differences between the wines from the different vintages were not statistically significant, even when considering wines from the same wineries in both vintages, which correspond to 22 wines out of the 32.

### Influence of Grape Origin, Withering, Grape Variety, and Vintage on DMS Formation during Aging

**Figure 2:**
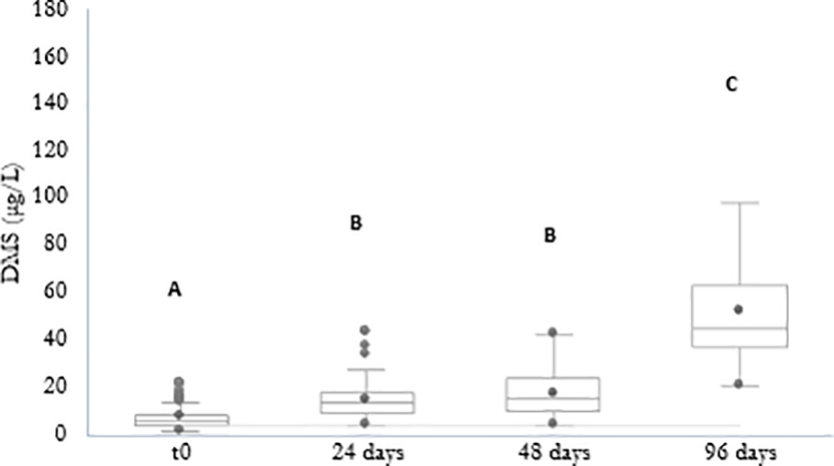
DMS (μg/L) at different time points of aging (0, 24, 48, and 96 days). Different letters denote statistically significant differences according to Kruskal–Wallis test (α = 0.05) with Dunn’s test (*p* < 0.05).

**Figure 3:**
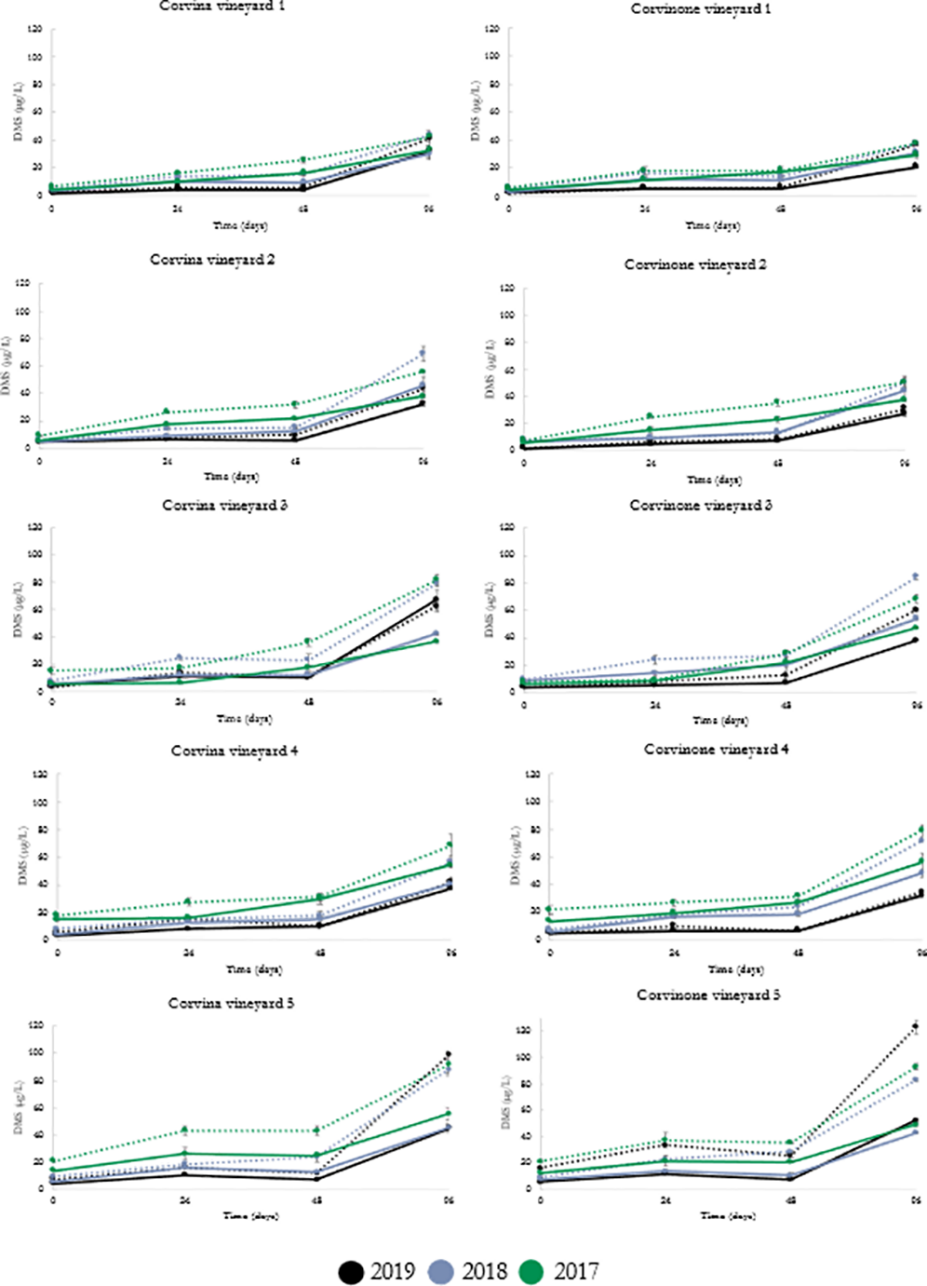
DMS concentrations (μg/L) during accelerated aging at 45 °C and different time points (0, 24, 48, and 96 days) of experimental wines (vintages 2017, 2018, and 2019) from five different vineyards. Continuous line (—), fresh; dotted line (---), withered.

**Figure 4:**
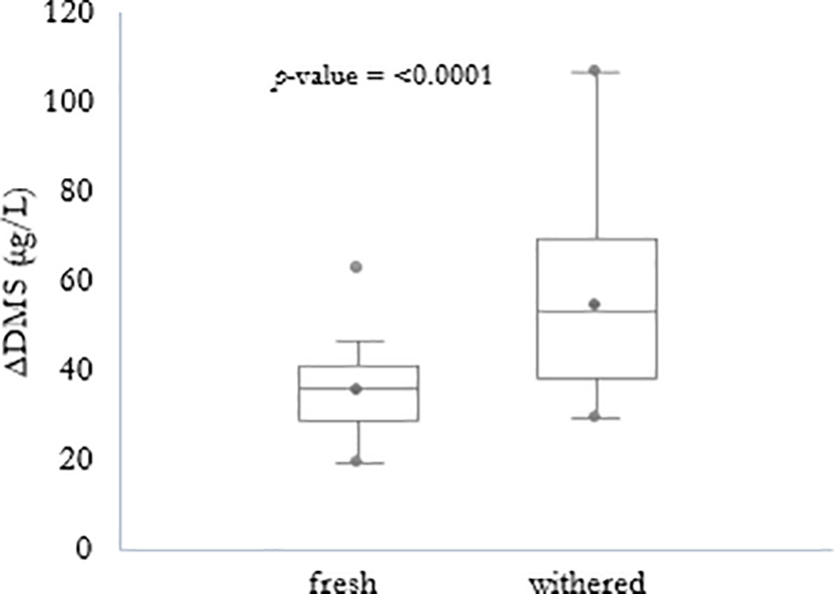
ΔDMS (μg/L) of fresh and withered wine samples, differences according to Kruskal–Wallis test (α = 0.05). ΔDMS = DMS at 96 days minus DMS before aging.

Figure 2 shows the concentration of DMS at three different time points during the accelerated aging of experimental wines obtained from either Corvina or Corvinone fresh and withered grapes coming from five different vineyards, whereas details on DMS concentrations at each time point can be found in Supporting Information S5. An initial (0–24 days) significant increase in DMS concentration was observed in all samples, whereas in the 24–48 days interval DMS remained stable. A much larger accumulation of DMS occurred then in the 48–96 days interval, with the highest concentrations surpassing 100 μg/L. Figure 3 provides a more detailed picture in which the influences of vineyard of origin, withering, and variety on DMS evolution during aging can be observed. Although the patterns of DMS accumulation were similar for all the wines, the vineyard of grape origin had a significant impact on DMS concentrations attained during aging. In particular, wines from vineyard 3 and vineyard 5 generally were the highest in DMS concentration at the end of aging, whereas wines from vineyard 1 produced less DMS with respect to all the others. Withering was also found to significantly influence DMS content, which increased in all cases in wines from withered grapes compared to that of fresh grapes (Figure 4).

Vintage was found to have a complex influence on DMS accumulation during aging, as statistically significant differences in DMS concentrations were observed only in the wines from certain vineyards and in certain vintages (Supporting Information S6). Conversely, variety did not influence significantly DMS content after aging (Supporting Information S7).

### Influence of pH on DMS Formation

Table 1 shows the results of the influence of pH on DMS formation in Amarone with and without SMM spiking. As expected, the highest DMS concentrations were found in samples added with SMM. At 1 week, we can observe an increase of around 10% in the spiked samples compared to the control samples. With respect to samples at one month, we can see a higher release of DMS on the spiked samples as expected. Instead, control samples showed lower concentrations of DMS: 43.1 μg/L (pH 3) and 51.5 μg/L (pH 4). Differences between spiked samples and control samples were statistically significant.

**Table 1:** Concentrations of DMS during Accelerated Aging at Different pH Levels^a^

sample	treatment applied	DMS released (μg/L)^b^	percentage (%) of conversion^c^
control	pH 3	19.4 (0.8)	
	1 week		
control	pH 4	23.4 (1.3)	
	1 week		
spiked^d^	pH 3	246.1 (7.8)	12
	1 week		
spiked^d^	pH 4	285.5 (7.6)	14
	1 week		
control	pH 3	43.1 (2.3)	
	1 month		
control	pH 4	51.5 (2.0)	
	1 month		
spiked^d^	pH 3	1016 (8.3)	49
	1 month		
spiked^d^	pH 4	1116 (11.3)	54
	1 month		

a Model aging was performed at 45 °C.

b In parentheses, standard deviation of DMS concentrations.

c Percentage (%) of conversion of the spiked samples was calculated as the ratio between the DMS released during aging and the theoretical releasable DMS (2064 μg/L).

d Spiked samples: dl-Methioninemethylsulfonium chloride was used as a precursor of DMS; 6450 μg/L was added, which corresponded to 2064 μg/L DMS.

## Discussion

The analysis of commercial Amarone wines revealed DMS presence at concentrations similar to those reported in other wines.^11,20,33−36^ Accurate comparisons of the data from different studies are however difficult due to the high variability in wine age at the time of analysis. Although some samples had relatively low DMS content, in nearly 60% of the samples, the DMS concentration was equal to or higher than the reported odor threshold of 27 μg/L in red wine. Overall, we can conclude that Amarone wines exhibit, at the time of their market release (which is minimum two years following the harvest year), DMS levels potentially impacting the wine perceived aroma based on the reported odor threshold. Another interesting observation related to Figure 1 is linked to the high variability observed for DMS in relation to wine producer, with differences up to a 10-fold factor in 2015. Segurel et al.^11^ reported in experimental Syrah and Grenache of the same vintage approximately 2-fold variations due to grape geographical origin variety as well as to variety, with Syrah being generally richer than Grenache. Grape variety was also shown to be an important factor by Fedrizzi et al.,^33^ whereas Le Menn et al.^34^ showed 2-fold variations in relationship to vineyard nitrogen status. In order to further investigate the factors associated with DMS variability in the context of Amarone, a set of experimental wines obtained from Corvina or Corvinone either fresh or withered grapes coming from five different vineyards was used. The results obtained (Figures 2 and 3) indicate that aging induced in all cases an increase in DMS wine content, in agreement with Segurel et al.,^2^ Fedrizzi et al.,^27^ and Ugliano et al.^12^ From a chemical point of view, this is due to the degradation of precursor compounds, among which *S*-methylmethionine (SMM) has been indicated as the most relevant one.^37^ Interestingly, the patterns reported in Figure 2 indicate, under our conditions, the existence of two distinct phases of DMS accumulation. In fact, an initial increase was observed in all wines, followed by a stationary phase and a second increase phase, which was typically more intense than the initial one. In most cases, this second phase of accumulation resulted in above threshold concentrations of DMS, with potentially relevant sensory implications associated with longer aging periods. The existence of this complex accumulation pattern might suggest the presence of different precursors having different reactivity. Segurel et al.^2^ highlighted the possibility that DMS formation could arise from chemical breakdown of compounds other than SMM, among which dimethyl sulfonium propanoic acid was considered but not confirmed. Cationic sulfur compounds were also indicated as a class of possible precursors DMS in beer, which would include SMM but also other compounds such as *S*-adenosylmethionine.^4^

While in all cases DMS concentration increased with accelerated aging, the extent of this process was influenced by various factors, among which vineyard of grape origin was found to be the most impactful. In a comparison between two vineyard sites during one vintage, vineyard-related variability in grape potential DMS was reported and was associated with grape nitrogen status and water deficit,^17^ whereas vintage was also reported to have a significant impact on DMS content of aged wines from the same vineyards.^18^ In the context of the present study, vineyard 3 and vineyard 5 resulted generally in wines with the highest in DMS concentration at the end of aging, whereas wines from vineyard 1 produced less DMS with respect to all the others. It is important to observe that these differences were generally minor and not significant in the wines prior to aging, so that it can be inferred that it was aging that allowed to reveal certain chemical differences associated with geographical origin of the wines. The fact that the association between vineyards of grape origin and highest/lowest postaging DMS was consistent across the three vintages indicates the existence of a “terroir” factor associated with DMS potential of single vineyard wines. Considering that vineyards 1–3 are located within an area of approximately 20 ha and vineyards 4 and 5 are located in other areas within Valpolicella, such terroir effect is likely to exist at the level of microscale, in agreement with previous observations for the Valpolicella region.^25^

In consideration of the previously reported relationship between vine nitrogen status and DMS potential,^17,18^ additional investigation was carried out on the potential relationship between DMS formed during aging and nitrogen parameters of enological relevance, such as for example primary amino nitrogen (PAN) content of grapes at crush. Additionally, as grape PAN is largely assimilated by yeast during fermentation, PAN content of the wines was also considered. Table 2 shows the results obtained from this correlation study considering the vineyard of origin, vintage, and variety. Overall, a positive correlation (Spearman correlation) was found in all samples between the amount of DMS produced and both wine and grape PAN, (ρ = 0.642 and ρ = 0.658, respectively). Strong correlations were also found by vineyard of origin and within wines of the same vintage. In some cases, however, either grape or wine PAN was not well-correlated with DMS formation. This is probably due to the complexity of DMS precursor metabolism during fermentation, as compounds such as SMM can be synthesized^38^ or degraded^14^ by yeast, with availability of other yeast assimilable nitrogen sources playing an important modulating role.^12^ The observation that PAN analysis can provide an estimate of the likelihood of different wines to form DMS during aging should be further explored as this could be of noticeable relevance in wine production. PAN analysis is commonly carried out in the winery to assess fermentation nutritional status of grape must, but its relevance to classification of aroma aging potential of finished wines has been little explored so far.

**Table 2:** Spearman’s Correlation Coefficients (ρ) between ΔDMS and PAN in Wines and Grapes^a^

	PAN in wines(mg/L)		PAN in grapes(mg/L)	
sample	Spearman ρ	*p*-value	Spearman ρ	*p*-value
wines vintage 2017	**0.666**	0.002	**0.632**	0.003
wines vintage 2018	**0.719**	0.001	**0.577**	0.009
wines vintage 2019	**0.657**	0.002	**0.741**	0.0002
wines vineyard 1	**0.713**	0.012	0.538	0.075
wines vineyard 2	**0.888**	<0.0001	**0.832**	0.001
wines vineyard 3	**0.664**	0.022	0.573	0.055
wines vineyard 4	0.399	0.201	**0.741**	0.008
wines vineyard 5	**0.867**	0.0004	0.389	0.213
Corvina wines	**0.608**	0.0004	**0.710**	<0.0001
Corvinone wines	**0.672**	<0.0001	**0.672**	<0.0001
all wines	**0.642**	<0.0001	**0.658**	<0.0001

a Values in **bold** correspond to significant correlation (Spearman, α = 0.05). ΔDMS was calculated as: DMS at 96 days – DMS concentration before aging.

Withering was also found to significantly influence DMS content, which increased in all cases in wines from withered grapes compared to fresh grapes (Figure 4). To our knowledge it is the first time that a relationship between grape withering and wine DMS content is described, although a general accumulation of amino acids due to dehydration in Corvina grapes has been recently reported.^39^ Also in our case, PAN of both grapes and wines was positively influenced by withering (Supporting Information S8). We hypothesize therefore that concentration of DMS precursors due to water loss is one major factor accounting for the higher DMS content of aged withered wines, although other possible factors will be addressed later in this study.

Conversely, vintage conditions were found to influence DMS in a complex way. First, in the case of commercial Amarone wines, no statistically significant influence of vintage was observed, which is probably reflecting the high variations associated with different geographical origin of the grapes as well as variable degrees and conditions of withering. The data on the aging of the experimental wines seem to confirm this, as comparison of individual vintages for each vineyard site and wine type indicated that vintage conditions could have a significant influence on DMS accumulation in wines from certain vineyard sites (Supporting Information S6). In the case of vineyards 1–3, all located in the town of Mezzane, 2018 was more frequently associated with increased DMS accumulation during aging, whereas in the case of vineyard 4 (located in San Pietro in Cariano) higher DMS accumulation was observed in both 2017 and 2019. Conversely, vintage 2019 was the one associated with increased DMS accumulation in wines from vineyard 5. Years 2017 and 2018 were generally warmer vintages compared to 2019, but the latter had much lower rainfall in the area of Marano, where vineyard 5 is located. These observations are in agreement with previous studies indicating that both temperature and water stress have been linked to increased DMS content of wines after aging^18,36^ but that more detailed studies should be carried out in relation to the influence of pedoclimatic conditions on DMS accumulation in Amarone.

### Influence of pH on DMS Production during Aging

Bekker et al.^16^ showed that pH influenced the release of DMS in Chardonnay and Shiraz wines. Higher pH has also been linked to somewhat higher DMS content in beer,^4^ and harsh alkaline conditions are widely being used to assess DMS-forming potential of wines.^2^ This positive association between DMS release and higher pH seems however more likely at pH higher than 5, while at lower pH, DMS formation from SMM is expected to take place through a nucleophilic substitution with water and so no influence of pH should be observed.^40^ In the model aging of Corvina and Corvinone wines described here, it was seen that withering had an influence on the production of DMS. Withering involves a partial dehydration that causes important chemical and physical modifications to the grape,^41^ including a significant increase in pH of finished wines (Supporting Information S9), in agreement with previous findings.^42^ Thus, the question arises as to which extent the pH increase associated with withering contribute to increased DMS formation. For this, the influence of pH on DMS formation during the aging of a Valpolicella wine with and without SMM spiking was investigated. Results (Table 1) confirm previous observations concerning the positive role of increased pH on DMS formation, as, in all cases, higher DMS released was observed at pH 4 compared to 3. However, considering that the pH range applied was quite broad for wine, it must be concluded that pH had a relatively limited contribution to the DMS increase associated with withering, with increases in the conversion rate of SMM to DMS in the 2–5% range. At a more general level, however, we can also conclude that DMS release from SMM can be modulated by pH even at values lower than 5 and therefore in wine conditions, possibly through degradation of SMM with formation of homoserine and DMS.^43,44^

In conclusion, this study highlighted the importance of DMS to the aroma composition of Amarone wines, in particular in relationship to cellar and bottle aging. DMS content of commercial Amarone is however highly variable, reflecting the complex influence of different production factors. Among these, the influence of the vineyard or origin was found to be of particular relevance, highlighting a relationship between DMS formation during aging and expression of terroir features. To this point, the observation of a strong correlation between wine (and to a good extent also grape) PAN content and DMS formation during aging is of great interest, as it indicates that the measurement of this parameter, already commonly carried out in the wine industry to assess fermentation nutritional status, can be used to assess the DMS forming potential of wines. This can be of relevance not only in terms of mapping vineyard characteristics but also in relationship to managing withering conditions and understanding the impact of vintage, as both these factors were observed to be important modulators of DMS content. Altogether, the results of this study provide useful clues in assisting winemakers to tailor DMS potential to specific Amarone styles, vineyard management procedures, and withering conditions.

## Supporting Information Available

Tables of main enological parameters of Amarone commercial wines, main characteristics of the vineyards regarding surface and soil, temperature and rainfall conditions of the three vintages, DMS concentrations and standard deviations of wines from three vintages, technological maturity of grapes at harvest, and statistical significance of differences in DMS produced during aging according to vintage and figures of ΔDMS of Corvina and Corvinone wines, concentrations of PAN in grapes and PAN in wines, and pH of wines made with fresh and withered Corvina and Corvinone grapes across three vintages (PDF)
