# Supplementary material for: Dimethyl Sulfide (DMS) in Amarone Wines: Influence of Aging, Withering, Grape Variety, and Geographical Origin
Source: J Agric Food Chem. 2023 Apr 21;72(4):1978–84. doi: 10.1021/acs.jafc.3c00728 (PMC10835709; doi:10.1021/acs.jafc.3c00728)
Supplement: Supplementary file 1 — jf3c00728_si_001.pdf [file jf3c00728_si_001.pdf]

Supporting information

-S1: Enological parameters of the commercial Amarone wines

-S2: Main characteristics of the vineyards

-S3: Temperature and rainfall conditions of the three vintages

-S4: Technological maturity of grapes at harvest

-S5: DMS concentrations of experimental wines from three vintages

-S6: Statistical significance of differences in DMS produced during aging according to vintage

-S7: Influence of grape variety on DMS formation during aging

-S8: Influence of withering on PAN content of grape and wines

-S9: pH of wines from fresh and withered grapes

S1: Enological parameters of the commercial Amarone wines

Table S1. Main enological parameters of Amarone commercial wines

Sample	*pH*	*% Alcohol*	*Residual sugars (g/L)*	*Acetic acid (g/L)*	*Free SO_2 (mg/L)*	*Total SO_2 (mg/L)*
**AM01**	3.39 (0.03)	15.50	4.4 (0.1)	0.45 (0.01)	11.1 (0.4)	39.0 (0.1)
**AM02**	3.54 (0.06)	15.00	6.6 (0.1)	0.49 (0.01)	19.5 (0.1)	65.5 (0.7)
**AM03**	3.45 (0.05)	16.00	2.8 (0.1)	0.69 (0.00)	15.8 (0.2)	56.0 (1.1)
**AM04**	3.45 (0.05)	15.00	7.4 (0.1)	0.53 (0.01)	15.0 (1.7)	49.0 (1.3)
**AM05**	3.33 (0.04)	15.50	3.0 (0.0)	0.60 (0.01)	7.5 (0.5)	46.0 (0.9)
**AM06**	3.41 (0.02)	16.00	5.6 (0.0)	0.36 (0.00)	20.0 (1.2)	104.0 (0.6)
**AM07**	3.37 (0.01)	15.00	1.4 (0.0)	0.61 (0.01)	36.2 (0.8)	70.5 (0.7)
**AM08**	3.33 (0.03)	15.50	6.4 (0.1)	0.50 (0.01)	30.9 (0.2)	59.0 (2.8)
**AM09**	3.39 (0.03)	16.50	4.7 (0.0)	0.65 (0.01)	28.6 (4.2)	45.0 (1.4)
**AM10**	3.32 (0.01)	16.50	0.1 (0.0)	1.01 (0.01)	36.9 (6.1)	61.0 (1.0)
**AM11**	3.57 (0.02)	16.00	7.8 (0.1)	0.51 (0.01)	11.5 (0.9)	36.5 (0.4)
**AM12**	3.06 (0.01)	16.50	1.5 (0.2)	0.49 (0.05)	10.9 (0.7)	39.5 (0.7)
**AM13**	3.09 (0.03)	16.50	2.6 (0.3)	0.82 (0.10)	11.0 (0.8)	35.2 (0.9)
**AM14**	3.27 (0.01)	15.00	4.3 (0.1)	0.58 (0.02)	16.1 (1.7)	45.0 (1.4)
**AM15**	3.28 (0.01)	15.00	7.3 (0.2)	0.49 (0.04)	27.7 (2.1)	76.5 (2.1)
**AM16**	3.31 (0.01)	15.00	8.4 (1.1)	0.61 (0.06)	34.2 (0.7)	78.0 (0.1)
**AM17**	3.27 (0.03)	15.00	4.3 (0.1)	0.78 (0.02)	18.9 (2.2)	44.0 (0.7)
**AM18**	3.43 (0.04)	15.00	3.8 (0.1)	0.75 (0.01)	11.6 (0.5)	74.0 (2.9)
**AM19**	3.58 (0.02)	15.00	4.6 (0.1)	0.48 (0.07)	15.4 (0.1)	111.0 (3.8)
**AM20**	3.39 (0.04)	16.00	4.8 (0.04)	0.54 (0.04)	8.8 (0.1)	85.0 (1.4)
**AM21**	3.45 (0.06)	16.50	2 (0.09)	0.58 (0.00)	7.2 (0.1)	89.0 (1.7)
**AM22**	3.49 (0.02)	15.50	3.8 (0.25)	0.93 (0.09)	5.6 (0.1)	88.0 (0.4)
**AM23**	3.54 (0.01)	14.50	2.7 (0.03)	0.41 (0.05)	11.0 (0.2)	133.0 (3.6)
**AM24**	3.57 (0.03)	16.00	3.6 (0.09)	0.55 (0.01)	3.3 (0.1)	92.0 (1.8)
**AM25**	3.38 (0.01)	15.50	7.0 (0.14)	0.58 (0.01)	2.5 (0.1)	97.0 (0.9)
**AM26**	3.55 (0.03)	16.00	2.6 (0.11)	0.57 (0.00)	2.60 (0.1)	58.0 (1.7)
**AM27**	3.42 (0.01)	15.50	2.9 (0.32)	0.61 (0.01)	1.8 (0.1)	73.0 (2.2)
**AM28**	3.45 (0.01)	15.00	5.7 (0.08)	0.45 (0.01)	24.8 (1.1)	161.0 (0.8)
**AM29**	3.41 (0.01)	15.00	6 (0.17)	0.39 (0.03)	19.8 (0.8)	128.0 (1.9)
**AM30**	3.46 (0.02)	15.00	3.9 (0.06)	0.49 (0.07)	5.4 (0.1)	86.0 (0.7)
**AM31**	3.48 (0.01)	16.50	4.2 (0.04)	0.57 (0.02)	9.6 (0.1)	83.0 (1.3)
**AM32**	3.43 (0.01)	15.00	2.5 (0.04)	0.59 (0.01)	7.5 (0.2)	95.0 (2.4)

S2. Main characteristics of the vineyards

Table S2. Main characteristics of the vineyards regarding surface and soil

Vineyard	Surface (ha)	Position and soil characteristics
V1	1	Slope 10-30%, terraced at 420-485 m a.s.l., south/south-west, sand35-40%, clay 20-25%, silt 35-45%
V2	3	Slope 20-30% at 290-345 m a.s.l., south/south-east, sand 40-45%, clay 25-30%, silt 25-35%
V3	3	Slope 30-35% at 270-370 m a.s.l., north/south-west, sand 35-45%, clay 20-30%, silt 25-45%
V4	2.5	Slope 70%, terraced at 150-195 m a.s.l., west, sand 45%, clay 20-25%, silt 30-35%
V5	7	Slope 30% at 430-500 m a.s.l., east/south-est, sand 46%, clay 17%, silt 38%

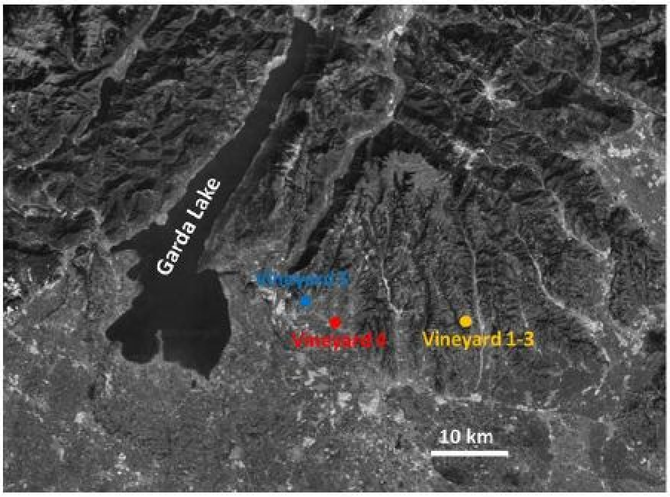

S3. Temperature and rainfall conditions of the three vintages

Table S3.1. Minimum, mean and maximum temperature (°C), expressed as monthly mean in the meteorological stations of Illasi, San Pietro in Cariano and Marano di Valpolicella

	Illasi			*San Pietro in Cariano*			*Marano di Valpolicella*		
	Min	Mean	Max	Min	Mean	Max	Min	Mean	Max
**2017**									
August	19.50	25.80	32.60	18.60	25.50	32.10	19.60	25.30	31.00
September	12.80	17.40	23.00	12.40	17.30	22.90	12.90	17.00	21.00
**2018**									
August	20.00	25.40	31.80	18.50	24.70	31.00	19.80	24.70	30.00
September	15.60	20.50	27.30	14.50	19.90	26.50	15.60	20.00	25.40
**2019**									
August	19.10	24.90	31.40	18.80	24.60	30.80	19.30	24.30	29.70
September	14.70	19.60	25.80	14.10	19.30	25.40	14.70	19.20	24.10

Table S3.2. Total rainfall (mm) and rainy days in the meteorological stations of Illasi, San Pietro in Cariano and Marano di Valpolicella

	Illasi		*San Pietro in Cariano*		*Marano di Valpolicella*	
	Rainfall (mm)	Days	Rainfall (mm)	Days	Rainfall (mm)	Days
**2017**						
August	16.6	4	24.2	2	31.8	2
September	108.0	12	95.2	13	146.8	13
**2018**						
August	42.6	6	97.2	9	87.2	9
September	239.4	7	171.4	5	188.2	5
**2019**						
August	52.8	4	82.2	7	65.8	7
September	85.2	6	106.8	5	62.0	4

S4: Technological maturity of grapes at harvest

Table S4.1. Sugar content (glucose + fructose g/L) and pH of fresh grapes at crush

	Glucose + fructose (g/L)		pH	
	average	SD	average	SD
**Corvina 2017**				
Vineyard 1	218.5	1.4	2.93	0.02
Vineyard 2	187.2	12.4	3.32	0.01
Vineyard 3	238.7	5.3	3.04	0.01
Vineyard 4	210.6	3.5	3.12	0.04
Vineyard 5	224.2	5.3	2.99	0.02
**Corvina 2018**				
Vineyard 1	202.0	1.7	2.97	0.02
Vineyard 2	183.0	4.4	3.10	0.01
Vineyard 3	233.7	4.0	3.03	0.03
Vineyard 4	213.7	2.5	3.18	0.01
Vineyard 5	201.7	2.5	2.87	0.03
**Corvina 2019**				
Vineyard 1	182.3	3.5	2.97	0.03
Vineyard 2	181.0	0.0	3.05	0.02
Vineyard 3	221.6	3.4	2.99	0.02
Vineyard 4	249.7	3.5	3.21	0.02
Vineyard 5	216.6	2.4	3.04	0.02
**Corvinone 2017**				
Vineyard 1	196.0	5.7	3.02	0.03
Vineyard 2	195.2	3.9	3.18	0.04
Vineyard 3	196.7	0.4	3.07	0.05
Vineyard 4	219.0	8.4	3.27	0.02
Vineyard 5	206.5	4.2	3.12	0.00
**Corvinone 2018**				
Vineyard 1	238.0	1.0	3.02	0.02
Vineyard 2	165.0	5.6	3.15	0.02
Vineyard 3	194.3	4.0	3.14	0.02
Vineyard 4	178.3	8.1	3.18	0.02
Vineyard 5	213.0	10.0	2.94	0.02
**Corvinone 2019**				
Vineyard 1	202.0	1.0	2.90	0.01
Vineyard 2	176.0	0.0	3.03	0.01
Vineyard 3	187.1	4.6	3.14	0.01
Vineyard 4	202.4	2.8	3.05	0.02
Vineyard 5	219.3	3.1	3.01	0.01

Table S4.2. Sugar content (glucose + fructose g/L) and pH of withered grapes at crush

	Glucose + fructose (g/L)		pH	
	average	SD	average	SD
**Corvina 2017**				
Vineyard 1	345.8	6.7	2.91	0.01
Vineyard 2	279.3	6.0	3.21	0.01
Vineyard 3	319.0	14.8	3.01	0.01
Vineyard 4	324.0	3.8	2.99	0.01
Vineyard 5	278.0	9.3	2.97	0.02
**Corvina 2018**				
Vineyard 1	255.6	19.1	3.01	0.01
Vineyard 2	253.3	7.0	3.06	0.03
Vineyard 3	272.3	10.8	3.00	0.02
Vineyard 4	305.3	2.8	3.21	0.02
Vineyard 5	262.4	1.5	3.02	0.02
**Corvina 2019**				
Vineyard 1	319.3	5.2	3.04	0.01
Vineyard 2	303.0	7.8	2.82	0.01
Vineyard 3	318.0	3.3	2.73	0.01
Vineyard 4	324.3	2.3	3.06	0.01
Vineyard 5	314.3	0.9	2.91	0.02
**Corvinone 2017**				
Vineyard 1	260.0	0.9	2.96	0.02
Vineyard 2	261.3	8.7	3.09	0.01
Vineyard 3	261.5	7.0	3.05	0.01
Vineyard 4	306.5	4.2	3.12	0.01
Vineyard 5	273.7	1.9	3.12	0.02
**Corvinone 2018**				
Vineyard 1	238.7	4.7	3.01	0.02
Vineyard 2	233.4	7.0	3.09	0.01
Vineyard 3	280.8	3.0	3.12	0.01
Vineyard 4	274.6	2.1	3.17	0.02
Vineyard 5	250.0	0.6	2.94	0.02
**Corvinone 2019**				
Vineyard 1	325.3	1.5	2.76	0.02
Vineyard 2	255.7	4.2	2.83	0.02
Vineyard 3	260.7	4.7	2.95	0.00
Vineyard 4	275.7	4.2	2.92	0.02
Vineyard 5	310.0	3.6	2.98	0.01

S5: DMS produced during aging according to vintage

Table S5.1. Concentration of DMS (μg/L)[1] of Corvina and Corvinone wines vintage 2017 analyzed in aging experiment.

Wines	*PAN wines (mg/L)*	*PAN grapes (mg/L)*	*Control*	*24 days*	*48 days*	*96 days*
V1-CA-F	5.4 (0.6)	49.0 (1.4)	4.2 (0.8)	9.8 (1.1)	16.4 (0.2)	32.5 (1.2)
V1-CA-W	29.1 (1.9)	53.0 (2.8)	6.2 (0.4)	15.8 (1.7)	25.4 (2.1)	41.9 (3.3)
V1-CO-F	5.7 (0.1)	37.5 (0.7)	4.8 (0.3)	11.7 (0.6)	17.1 (1.7)	29.5 (2.3)
V1-CO-W	12.9 (1.1)	40.0 (3.5)	6.0 (1.2)	18.0 (3.1)	18.2 (3.1)	37.4 (1.4)
V2-CA-F	13.7 (1.0)	105.5 (2.1)	5.1 (0.3)	17.1 (1.5)	21.5 (1.0)	37.7 (0.8)
V2-CA-W	32.2 (1.7)	156.5 (0.7)	9.1 (0.2)	26.3 (0.4)	31.6 (2.3)	55.6 (1.0)
V2-CO-F	11.8 (1.4)	70.5 (4.9)	5.8 (0.9)	15.1 (2.7)	23.1 (2.4)	37.4 (0.8)
V2-CO-W	25.1 (0.8)	82.0 (3.6)	8.0 (1.5)	25.0 (0.1)	35.3 (2.5)	50.5 (3.9)
V3-CA-F	8.1 (0.0)	43.5 (0.7)	5.3 (0.8)	6.2 (0.8)	17.2 (1.8)	36.6 (0.9)
V3-CA-W	20.8 (1.9)	93.5 (3.5)	15.0 (2.9)	17.2 (1.3)	35.9 (2.7)	81.6 (4.8)
V3-CO-F	9.0 (1.1)	49.0 (4.2)	6.4 (0.9)	8.5 (0.1)	22.0 (3.3)	47.4 (0.7)
V3-CO-W	26.6 (0.2)	70.3 (1.7)	8.0 (1.5)	9.5 (1.5)	28.9 (2.1)	68.5 (3.5)
V4-CA-F	8.7 (0.4)	64.3 (5.0)	14.9 (1.0)	16.2 (1.2)	29.9 (4.0)	54.3 (1.4)
V4-CA-W	16.9 (3.8)	63.0 (2.0)	18.0 (0.4)	27.4 (2.5)	31.1 (2.6)	68.8 (9.0)
V4-CO-F	11.9 (1.5)	77.3 (4.0)	13.4 (0.5)	19.4 (2.8)	26.4 (1.4)	56.3 (6.5)
V4-CO-W	15.5 (0.6)	54.3 (5.0)	21.6 (3.5)	26.8 (2.5)	31.2 (2.3)	78.9 (4.0)
V5-CA-F	8.4 (1.0)	78.0 (2.8)	13.9 (0.6)	26.2 (4.4)	24.9 (0.4)	55.6 (4.3)
V5-CA-W	29.8 (1.9)	131.0 (3.5)	20.9 (0.3)	43.0 (3.5)	42.8 (3.5)	91.3 (6.3)
V5-CO-F	5.2 (0.7)	63.5 (2.1)	12.5 (0.5)	21.4 (4.2)	20.7 (0.9)	48.8 (0.9)
V5-CO-W	24.5 (1.2)	81.7 (5.3)	21.1 (1.3)	37.2 (6.0)	35.5 (0.9)	92.6 (2.4)

[1]In parenthesis standard deviation of analytical replicates. [2]Codes use for wines: CA= Corvina, CO= Corvinone, F= fresh, W= withering.

Table S5.2. Concentration of DMS (μg/L)[1] of Corvina and Corvinone wines vintage 2018 analyzed in aging experiment.

Wine codes[2]	*PAN wines (mg/L)*	*PAN grapes (mg/L)*	*Control*	*24 days*	*48 days*	*96 days*
V1-CA-F	4.9 (0.3)	55.7 (0.6)	3.1 (0.1)	10.2 (0.4)	9.1 (1.2)	29.7 (4.2)
V1-CA-W	21.9 (0.7)	64.7 (2.6)	5.1 (0.1)	13.1 (0.2)	15.3 (1.5)	43.4 (2.8)
V1-CO-F	14.1 (0.2)	65.7 (6.4)	3.2 (0.1)	12.1 (1.1)	11.4 (0.5)	31.3 (0.7)
V1-CO-W	25.9 (0.4)	94.0 (7.9)	5.7 (0.1)	16.8 (1.7)	13.9 (0.6)	37.6 (0.9)
V2-CA-F	28.4 (1.1)	102.3 (8.7)	5.1 (0.7)	9.0 (1.1)	12.1 (2.3)	45.9 (6.3)
V2-CA-W	48.8 (1.1)	107.8 (7.5)	5.7 (0.1)	13.7 (0.6)	15.3 (1.0)	68.8 (5.1)
V2-CO-F	21.9 (0.8)	81.3 (4.6)	7.2 (0.5)	6.9 (0.5)	13.6 (0.4)	44.7 (6.6)
V2-CO-W	29.7 (1.1)	113.0 (1.7)	8.0 (0.1)	8.0 (1.4)	13.1 (0.2)	50.5 (4.1)
V3-CA-F	16.1 (0.1)	105.7 (2.5)	4.5 (0.2)	11.9 (0.1)	11.8 (1.8)	42.4 (0.9)
V3-CA-W	48.1 (1.1)	160.3 (3.5)	7.2 (0.1)	24.3 (1.0)	22.9 (3.9)	79.1 (1.2)
V3-CO-F	29.2 (0.6)	120.7 (1.2)	8.2 (0.1)	14.2 (1.8)	20.2 (3.6)	54.4 (1.3)
V3-CO-W	57.5 (3.2)	153.3 (2.7)	9.3 (0.2)	24.2 (3.0)	27.1 (3.4)	84.6 (1.9)
V4-CA-F	10.3 (0.2)	56.3 (1.5)	3.7 (0.4)	12.7 (0.2)	15.1 (2.4)	40.7 (1.8)
V4-CA-W	22.3 (3.5)	70.7 (5.7)	7.4 (0.2)	15.0 (2.1)	17.5 (2.9)	56.7 (3.0)
V4-CO-F	13.4 (0.4)	54.7 (1.5)	5.1 (0.1)	16.3 (0.2)	18.7 (1.6)	48.2 (2.6)
V4-CO-W	25.8 (0.9)	90.0 (0.8)	6.7 (0.4)	18.2 (0.8)	23.7 (3.8)	71.2 (0.5)
V5-CA-F	14.0 (0.2)	69.7 (2.1)	5.6 (0.5)	16.1 (0.1)	12.6 (1.3)	45.1 (2.0)
V5-CA-W	26.8 (0.7)	114.7 (5.0)	9.5 (1.8)	18.5 (1.1)	23.6 (3.1)	87.9 (3.9)
V5-CO-F	18.3 (0.1)	104.3 (0.6)	7.7 (0.3)	13.9 (1.0)	10.5 (2.0)	42.9 (0.9)
V5-CO-W	31.7 (4.9)	95.7 (6.5)	9.2 (0.6)	22.9 (2.4)	28.6 (1.4)	82.9 (1.1)

[1]In parenthesis standard deviation of analytical replicates. [2]Codes use for wines: CA= Corvina, CO= Corvinone, F= fresh, W= withering.

Table S5.3. Concentration of DMS (µg/L)[1] of Corvina and Corvinone wines vintage 2019 analyzed in aging experiment.

Wine codes[2]	*PAN wines (mg/L)*	*PAN grapes (mg/L)*	*Control*	*24 days*	*48 days*	*96 days*
V1-CA-F	4.3 (0.1)	28.7 (3.5)	1.6 (0.2)	4.0 (0.5)	3.8 (0.1)	31.8 (4.00)
V1-CA-W	49.8 (1.0)	76.1 (3.5)	2.6 (0.2)	6.0 (0.8)	6.0 (0.6)	40.7 (1.5)
V1-CO-F	12.2 (0.7)	37.7 (1.7)	2.5 (0.2)	5.4 (0.6)	5.8 (0.4)	21.1 (0.4)
V1-CO-W	23.7 (0.2)	82.7 (4.0)	2.0 (0.1)	5.4 (0.8)	6.6 (0.2)	37.1 (0.2)
V2-CA-F	12.6 (0.2)	49.3 (4.7)	4.5 (0.2)	6.3 (0.2)	5.8 (0.5)	32.1 (1.8)
V2-CA-W	32.9 (0.1)	121.1 (4.0)	5.3 (0.2)	7.1 (0.4)	9.4 (0.9)	43.4 (2.4)
V2-CO-F	8.0 (0.0)	42.3 (1.5)	1.7 (0.1)	4.9 (0.1)	7.4 (0.9)	27.9 (2.1)
V2-CO-W	20.9 (0.1)	44.7 (1.8)	1.9 (0.1)	6.7 (0.1)	9.00 (1.5)	30.9 (0.6)
V3-CA-F	11.9 (0.3)	73.3 (6.4)	4.4 (0.2)	10.6 (0.3)	10.1 (0.4)	67.2 (9.2)
V3-CA-W	36.1 (1.1)	68.1 (4.3)	2.9 (0.3)	13.5 (2.2)	11.1 (0.1)	62.0 (3.4)
V3-CO-F	17.1 (0.7)	70.5 (7.2)	4.3 (0.1)	5.3 (0.5)	7.3 (0.4)	38.2 (0.4)
V3-CO-W	38.0 (1.0)	77.4 (0.7)	3.9 (0.1)	8.3 (0.3)	12.6 (0.2)	60.6 (1.1)
V4-CA-F	9.6 (0.1)	45.9 (3.1)	3.4 (0.1)	8.2 (0.4)	9.9 (0.5)	37.8 (1.1)
V4-CA-W	29.4 (0.5)	44.5 (1.2)	5.6 (0.1)	14.9 (0.5)	10.2 (0.5)	41.9 (3.2)
V4-CO-F	9.6 (0.1)	45.4 (2.8)	4.9 (0.1)	6.4 (0.1)	6.7 (0.6)	31.9 (0.1)
V4-CO-W	20.1 (0.2)	43.3 (2.2)	4.7 (0.4)	10.1 (1.5)	6.3 (0.4)	34.6 (1.3)
V5-CA-F	14.7 (0.2)	105.7 (2.9)	4.4 (0.2)	10.8 (0.2)	7.3 (0.9)	44.7 (3.4)
V5-CA-W	90.8 (3.7)	114.7 (3.5)	7.4 (0.2)	16.1 (0.7)	11.8 (0.2)	98.4 (2.9)
V5-CO-F	19.8 (0.7)	99.8 (5.4)	5.4 (0.3)	11.7 (0.9)	7.5 (1.0)	52.1 (1.2)
V5-CO-W	53.2 (1.0)	98.4 (11.8)	16.0 (0.2)	33.5 (0.3)	28.4 (1.0)	122 (7.6)

[1]In parenthesis standard deviation of analytical replicates. [2]Codes use for wines: CA= Corvina, CO= Corvinone, F= fresh, W= withering.

S6: Statistical significance of differences in DMS produced during aging according to vintage

Table S6. Significant differences between DMS concentrations at 0, 24, 48, 96 days and ΔDMS of vintages 2017, 2018 and 2019 according to Kruskal-Wallis test (α=0.05)

	0 days			24 days			48 days			96 days			ΔDMS		
	2017-2018	2017-2019	2018-2019	2017-2018	2017-2019	2018-2019	2017-2018	2017-2019	2018-2019	2017-2018	2017-2019	2018-2019	2017-2018	2017-2019	2018-2019
Fresh grapes															
Vineyard 1	0.117	**0.002**	0.117	0.845	**0.024**	**0.014**	0.117	**0.002**	0.117	**0.845**	0.327	0.433	0.556	0.769	0.377
Vineyard 2	0.556	**0.039**	**0.008**	0.117	**0.002**	0.117	0.117	**0.002**	0.117	0.117	0.117	**0.002**	0.170	0.096	**0.002**
Vineyard 3	0.845	**0.031**	**0.050**	**0.016**	0.922	**0.021**	0.433	**0.006**	**0.050**	0.239	0.239	0.976	0.239	0.239	0.976
Vineyard 4	**0.039**	**0.008**	0.556	0.239	**0.003**	0.078	0.117	**0.002**	0.117	0.117	0.002	0.117	1.000	**0.019**	**0.019**
Vineyard 5	0.096	**0.002**	0.170	0.117	**0.002**	0.117	0.117	**0.002**	0.117	**0.019**	0.377	0.141	0.492	0.202	**0.050**
Withered grapes															
Vineyard 1	0.239	**0.003**	0.239	0.433	**0.006**	**0.050**	0.170	**0.002**	0.096	0.731	0.961	0.694	0.624	0.202	0.433
Vineyard 2	0.239	**0.003**	0.078	0.096	**0.002**	0.170	0.117	**0.002**	0.117	0.695	**0.031**	**0.011**	0.695	**0.031**	**0.011**
Vineyard 3	0.695	**0.011**	**0.031**	**0.039**	0.556	**0.008**	0.170	**0.002**	0.096	0.327	0.062	**0.004**	0.117	0.202	**0.004**
Vineyard 4	0.117	**0.002**	0.117	0.078	**0.003**	0.239	0.117	**0.002**	0.117	0.239	**0.003**	0.078	0.695	**0.031**	**0.011**
Vineyard 5	**0.019**	**0.019**	1.000	**0.031**	**0.050**	0.845	0.062	**0.004**	0.327	0.170	0.096	**0.002**	0.239	**0.003**	0.078

S7. Influence of grape variety on DMS produced during aging

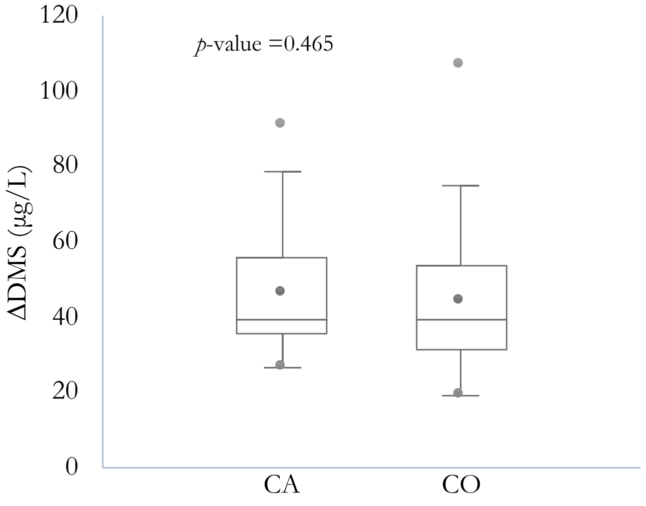

Figure S7. ΔDMS (μg/L) of Corvina (CA) and Corvinone (CO) wines, *p*-value according to Kruskal-Wallis test (α=0.05). ΔDMS = DMS at 96 days minus DMS before aging.

S8: Influence of withering on PAN content of grapes and wines

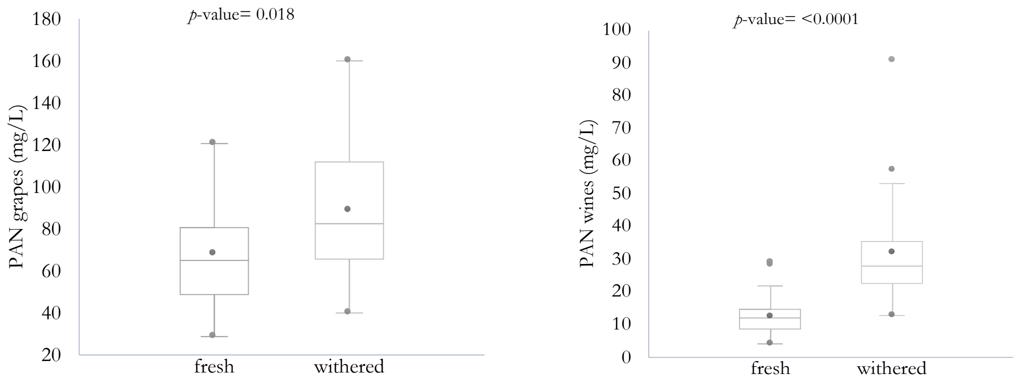

Figure S8. Concentrations of a) PAN in grapes (mg/L) and b) PAN in wines (mg/L) from fresh and withered samples, *p*-value according to Kruskal-Wallis's test (α=0.05).

S9: Influence of withering on wine pH

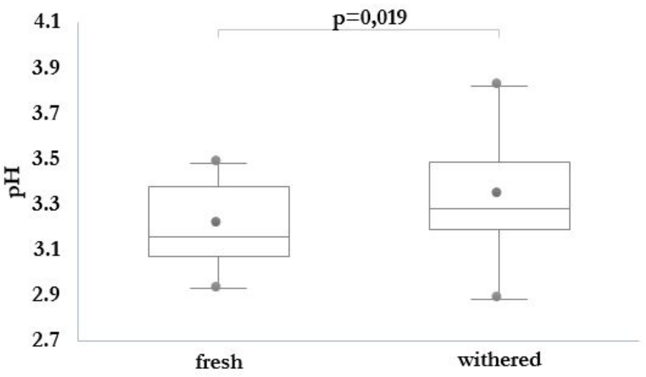

Figure S9. pH of wines made with fresh and withered Corvina and Corvinone grapes across three vintages and belonging to five different vineyards, statistically different according to Kruskal-Wallis's test (α=0.05).
